# Electronic structure of the PrNiBi half-Heusler system based on the *σ*GGA + U method

**DOI:** 10.1038/s41598-019-56537-1

**Published:** 2019-12-27

**Authors:** L. Mikaeilzadeh, A. Tavana, F. Khoeini

**Affiliations:** 1 Department of Physics, University of Zanjan, Zanjan, 45195-313 Iran; 2 AMDM Lab., Department of Physics, University of Mohaghegh Ardabili, Ardabil, 179 Iran

**Keywords:** Materials science, Nanoscience and technology, Magnetic properties and materials

## Abstract

In this works, we study the electronic structure and magnetic properties of the Pr-Ni-Bi half-Heusler systems based on density functional theory. We use the *σ* GGA + U scheme to consider the effects of on-site electron-electron interactions. Results show that in contrast to the rough estimation of the total magnetic moment of the unit cell, based on the Slater-Pauling behavior in the half-Heusler systems, this system has an antiferromagnetic ground state because of the localized Pr-f electrons. By increasing the magnitude of the effective U parameter, band-inversion occurs in the band structure of this system, which shows the possibility of topological state occurrence.

## Introduction

Full/half-Heusler compounds with chemical formula *X*_2_*YZ*/*XYZ* are very attractive due to their interesting physical properties resulting from their tunable crystal structures^1–15^. In their chemical formula, X and Y are transition metal or rare earth (RE) elements and Z is a sp element. When X or Y is a RE element, half-Heusler compounds usually attract much more attention because of the emerging novel physical properties, e.g. superconductivity (SC)^16–20^, thermoelectric behavior (TE)^21–24^, various magnetic orderings^18,19,23,25,26^, topological band properties^19,27–30^, etc.^31–33^.

The topological insulating state is one of the most important categories of topological phases in condensed matter systems. Highly non-trivial transport properties of this electronic state can be used in novel electronic devices^34,35^. The concept of topological insulators is originated from the discovery of the quantum spin Hall effect^36–38^ in systems that are insulator in bulk while having time reversal symmetry-protected surface states, located in the bulk energy gap. The HgTe compound is the most studied topological insulator (TI), that has a non-trivial s-p band inversion in its band structure^39,40^. Theoretical studies on Heusler alloy TIs has begun in the recent decade. Investigations indicated that compounds such as LnPtBi, LnPdBi and LnAuPb in their nonmagnetic phases show s-p band inversion at the time reversal-invariant momentum, i.e. the Γ point, similar to HgTe^41–45^. In recent years, this non-trivial behavior are confirmed by NMR and ARPES experiments^28,46–48^.

On the other hand, empirical calculations suggest that some of these compounds have SC or antiferromagnetic (AFM) ground states. Coexistence of the topological state with these phases is interesting and significant. In 2010, Mong introduced the concept of antiferromagnetic topological insulator (AFTI)^49^. The main feature of TIs in nonmagnetic phases is the symmetry-protected-surface states while in AFM phase this symmetry is broken. In order to preserve the Kramer’s degeneracy and be able to define the topological invariant index, *Z*_2_, the system must have some degrees of symmetry in the AFM state. This symmetry is the combined symmetry of time reversal and the lattice translation vector, **D**, which preserves the spin order^49,50^. GdPtBi and NdPtBi compounds which are AFM at low temperatures with, respectively, orthorhombic and tetragonal magnetic structures are the first known AFTI compounds^51–53^.

In this paper, we investigate the electronic and magnetic properties of the PrNiBi compound. Hasse *et al*. have synthesized this compound for the first time in 2002^54^. Experimental investigations show this compound has an AFM order at temperatures below 10 K^55^. Measurements of the magnetic susceptibility shows the Curie-Weiss temperature behavior in this compound at high temperatures with an anomaly at low temperatures which proves the AFM ordering. Resistivity measurements indicate metallic behavior at temperatures above the Neel temperature (*T*_*N*_) and semiconducting behavior in the AFM phase. So, in order to study the ground state band structure of this compound, first, we study the PrNiBi compound, without considering spin polarization. Following that, we perform spin-polarized calculations in which the on-site 4f-electron interactions are taken into account based on the *σ*GGA + U scheme. Finally, we investigate two possible AFM configurations of this system.

## Computational Details

Band structure calculations are performed using the full-potential linearized augmented plane-waves (FP-LAPW) approach of density functional theory (DFT), as is implemented in the Wien2k code^56^. The generalized gradient approximation (GGA) is used for the exchange-correlation functional. Converged ground states are obtained by using the cut-off value of 9 for the expansion of wave functions, *R*_*MT*_ × *K*_*MAX*_, where *R*_*MT*_ is the smallest muffin-tin radius in the unit cell and *K*_*MAX*_ is the magnitude of the largest K-vector. The muffin-tin radii for Ni, Pr, Bi are set equal to 2.5, 2.55 and 2.6 a.u., respectively. For the cubic structure, calculations are performed on a 12 × 12 × 12 k-point mesh and for the orthorhombic and the tetragonal magnetic structures, on 9 × 9 × 9 and 7 × 10 × 10 k-point meshes, respectively, constructed based on the Monkhorst-Pack scheme. The k-meshes are selected so that the density of k-points are almost the same in all of the calculations. The convergence criterion for energy and charge were set equal to 10^−4^ *Ry* and 0.01*e*, respectively. Equilibrium lattice parameters in all calculations are obtained by fitting the volume-energy plots to the Murunaghan equation of state. All the parameters of the calculations are carefully checked to be converged.

The *σ*GGA + U method^57^ is used to account for the on-site interactions at the Pr sites. This adds an orbital dependent correction to the intra-atomic Coulomb and exchange potentials which is underestimated in ordinary GGA calculations. Here, the effective Coulomb-exchange interaction, *U*_*eff*_, is the difference between the Hubbard-U and exchange-J interactions, i.e. *U*_*eff*_ = *U* − *J*. In the rest of the paper we always work with this effective value. Particularly, the self-interaction correction (SIC) scheme is used to incorporate the double-counting correction, 1/2*UN*(*N* − 1), which is favorable for the strongly correlated electron system.

The value of the U parameter should be determined empirically and is sensitive to the details of the computational method. A previous evaluation of the U parameter for the f-orbitals of the Pr atom in a relatively similar environment and with the same computational approach have yield the value of 0.74 Ry.^58^ So, in this work the range of the U parameter is selected according to this value. We have also investigated the effect of spin orbit coupling (SOC) on band structures, which is crucial in spin quantum Hall systems.

## Results and Discussion

### Structural properties

**Figure 1:**
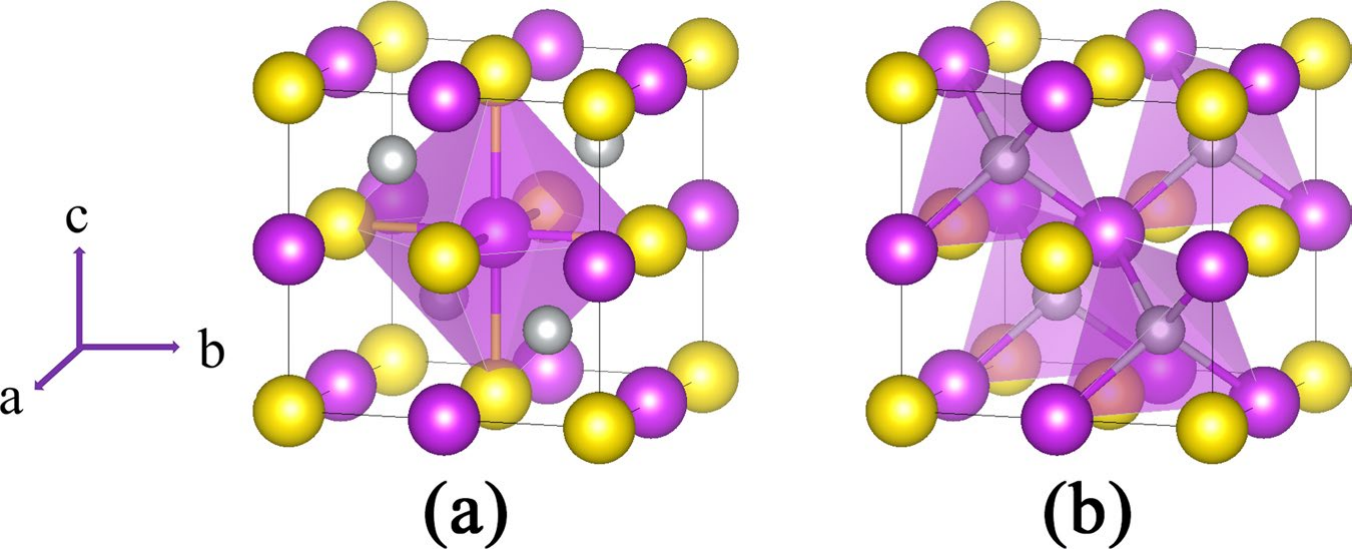
Crystal structure of PrNiBi compound distinguishing the octahedral (**a**) and tetrahedral (**b**) positions. Yellow, purple and gray balls represent Pr, Bi and Ni atoms, respectively.

Half-Heusler XYZ compounds have a non-centrosymmetric cubic structure with $F\bar{4}3m$ space group, where X and Y are transition metal or RE elements, and Z is a main group III-VI element. In Fig. 1, the crystal structure of the PrNiBi is depicted as is obtained from the XRD experiment^55^. As depicted in Fig. 1, Y and Z atoms are located at octahedral 4*a* and 4*b* Wyckoff positions, i.e. (0,0,0) and (1/2,1/2,1/2), and X atoms occupy tetrahedral 4*c* positions, i.e. (1/4,1/4,1/4). X and Z atoms with largest electro-negativity difference usually sit on a NaCl-like sub-lattice, while Y and Z atoms are covalently bonded on a ZnS-like sub-lattice.

### Non-spin polarized calculation

**Figure 2:**
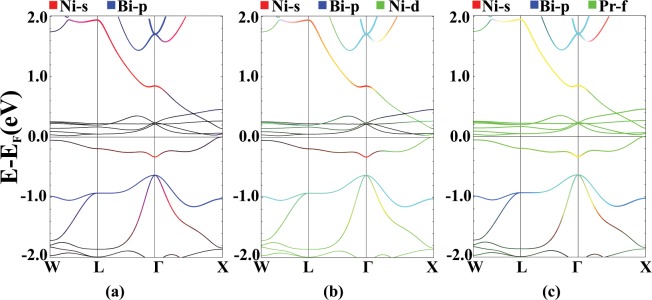
Non-spin polarized band structure of the PrNiBi with Ni-s (red), Bi-p (blue), Pr-f (green) and Ni-d (green) characterization. Calculation are performed for relaxed lattice parameters.

We start with non-spin polarized (NSP) calculation for the PrNiBi compound. The equilibrium lattice parameter is obtained equal to 6.58 Å, which is almost one percent larger than the experimental value of 6.508 Å^55^. This is reasonable because the GGA approximation usually overestimates the experimental lattice parameters. The calculated band structure is shown in Fig. 2. As it is clear from the figure, six Pr-f orbital bands are located in the energy range −0.4 to 0.7 eV respect to the Fermi level which are hybridized with the Ni-d states. Parts of the Pr-f states appear 0.7 to 2 eV above the Fermi level with strong hybridization with the Ni-s orbital at the Γ point. The remaining Pr-f states are hybridized with the Bi-p states at the Γ point at energies −0.8 eV below the Fermi level. The hybridization of the Pr-f and the Bi-p at the Γ point indicates the ionic nature of their interaction, as is expected. On the other hand, hybridization of the Pr and the Ni states indicates the formation of covalent bonding between these elements. Strong hybridization between Ni-d and Bi-p states can be observed below the Fermi level, which shows the strong coupling between these atoms.

### Spin-polarized calculation

In the continuation, calculations are performed for the spin-polarized ferromagnetic (FM) state. In the study of topological phases in half-Heusler alloys, many DFT calculations have only investigated the non-spin polarized states even in the presence of magnetic or RE elements^27,41,42^. This has been done, so far, because of a rough argument based on the Slater-Pauling (SP) behavior in half-Heusler alloys. According to this argument, in half-Heusler compounds, the average magnetic moment per atom is equal to zero if the number of valence electrons per formula unit is equal to 18. In the case that the compound consists of a RE element, it is supposed that the f electrons occupy the core state. Hence, based on a rough enumeration of other valence electrons, the total magnetic moment of the unit cell is considered to be zero. However, it is noticeable that the SP behavior is only applicable to the system in which the magnetism arises from the itinerant electrons. In the PrNiBi system, considering the Pr-f electrons as core states leads to zero average magnetic moments for every atom, based on the SP reasoning. However, This is in obvious contradiction with the initial assessments, making it inevitable that spin-polarized calculations should be performed.

In spin-polarized FM calculations, the equilibrium lattice parameter is obtained equal to 6.6443 Å, which is two percent larger than the experimental lattice parameter. Total energy of the FM state is 0.6 eV lower than the NSP state. it shows that the FM phase of the PrNiBi compound is more favorable in comparison with the paramagnetic state.

**Figure 3:**
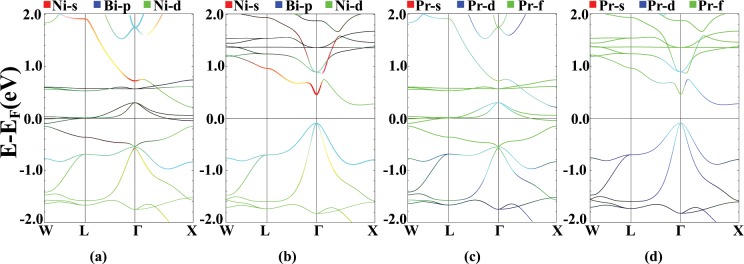
FM band structure of the PrNiBi in spin-majority (**a**,**c**) and spin-minority (**b,d**) channels with Ni-s (red), Bi-p (blue), Ni-d (green), Pr-s (red), Pr-d (blue) and Pr-f (green) characterization. Calculation are performed for relaxed lattice parameters.

Results of the FM band structure calculations are shown in Fig. 3, indicating a half-metallic behavior^59,60^, i.e metallic in the spin majority channel and semiconducting in the spin minority channel with a 0.4 eV indirect energy gap. In the spin majority channel atomic orbital characterizations show that similar to the NSP case, most of the f orbital states pin to the Fermi level in the energy range of −0.5 to 0.5 eV, showing noticeable mixture with the Ni-d orbitals. Similarly, remaining Pr-f states are hybridized with the Ni-s orbitals, 0.7 eV above the Fermi level and with the Bi-p orbitals, 0.5 eV below the Fermi level.

### *σ*GGA + U calculations

There are few spin-polarized calculations performed for half-Heusler alloys which explore the effect of on-site electron interactions on the band structure. Fermi level pinning of the f states is the well-known deficiency of ordinary exchange-correlation functionals that can be corrected, considerably, by including the on-site Coulomb interaction between the localized electrons, in strongly correlated electron systems. This is, practically, done by adding the orbital-dependent Hubbard-U potential to the exchange-correlation energy. In our calculations, the Hubbard potential is considered based on the self interaction correction (SIC) scheme, which is more suitable for the strongly correlated systems. Calculations are performed with different effective values of U up to 1.2 Ry.

**Figure 4:**
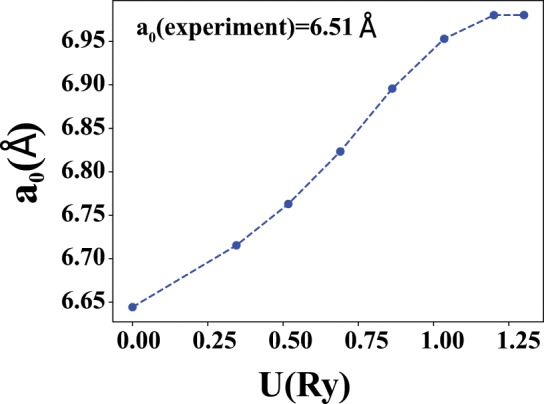
U dependence of the equilibrium lattice parameter, from spin polarized volume optimization calculations.

The obtained equilibrium lattice parameters are plotted in Fig. 4 as a function of U. As it is clear from the figure, the equilibrium lattice parameter increases by increasing the Hubbard U up to the value of 1 Ry, after which it does not change considerably. This behavior is due to the energy shift of the Pr-f states which affects the electronic structure.

**Figure 5:**
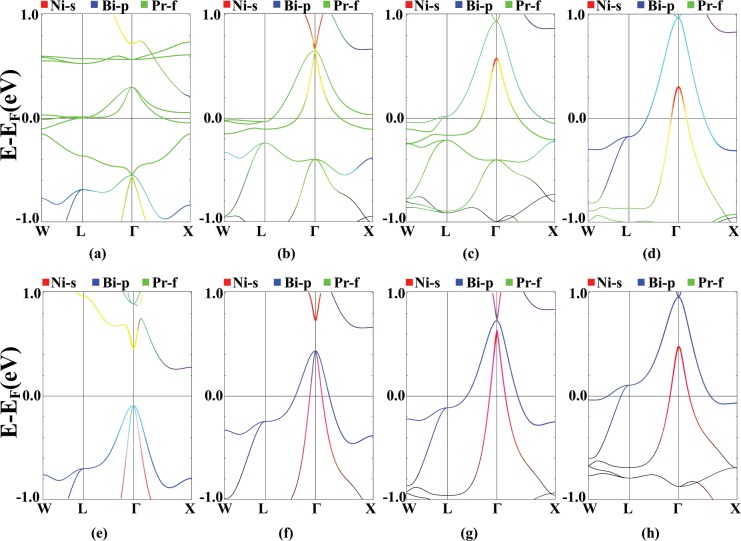
FM, *σ*GGA + U band structures for different values of U = 0, 0.345, 0.69 and 1.035 Ry in majority ((**a**–**d**)) and minority ((**e**–**h**)) spin channels, with Ni-s (red), Bi-p (blue) and Pr-f (green) states characterization. Calculation are performed for relaxed lattice parameters obtained for each U value.

Results of band structure calculations for different effective values of U are plotted in Fig. 5. In the majority spin channel, by increasing the U value, the Pr-f states, that were pinned to the Fermi level in GGA calculations, are pushed toward lower energies and the strong hybridization of the Pr-f states with Ni-s and Bi-p states is substantially broken. This causes the Bi-p bands to form at higher energies which, in turn, leads to Ni-s-Bi-p band inversion, for the U values larger than 0.345 Ry. In the minority spin channel, by increasing U, the Bi-p states are pushed above the Fermi level and the band gap closes for U values larger than 0.69 Ry. In this channel, the Pr-f states appear at high energy values with almost no hybridization with other atoms.

**Figure 6:**
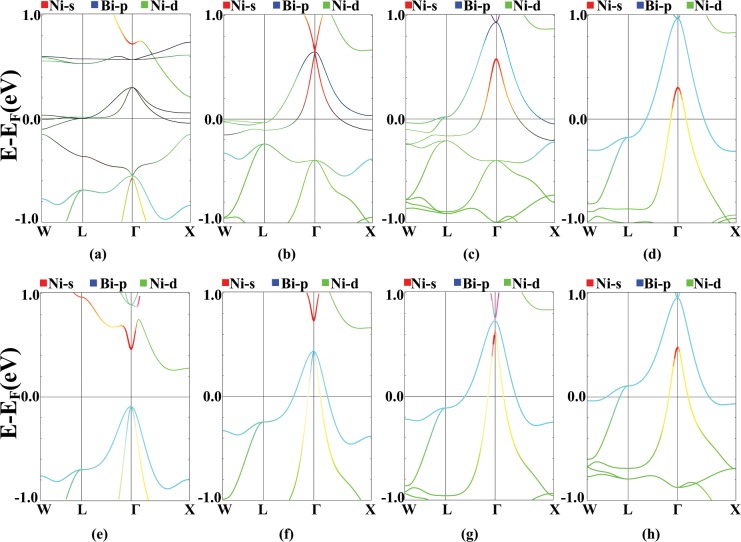
FM, *σ*GGA + U band structures for different values of U = 0, 0.345, 0.69 and 1.035 Ry in majority ((**a**–**d**)) and minority ((**e**–**h**)) spin channels, with Ni-s (red), Bi-p (blue) and Ni-d (green) orbitals characterization. Calculation are performed for relaxed lattice parameters obtained for each U value.

In Fig. 6, the contribution of Ni-d states in different energy bands is characterized. In the spin majority channel, most of the Ni-d bands form below the Fermi level. By increasing the value of U, there is a competition between Pr-f and Ni-d states in hybridizing with the Bi-p states at the Fermi energy. For large values of U, the bonding between the Pr-f and other states breaks and a strong hybridization occurs between Ni and Bi atoms at the Γ point, 0.9 eV above the Fermi level. In the minority spin channel, because of the absence of Pr-f states, this hybridization is not affected by applying U. In all of the band structure diagrams, the Pr-d orbitals are strongly mixed with the Bi-p states and by applying the Hubbard U, Ni-d orbitals also mix with them.

**Figure 7:**
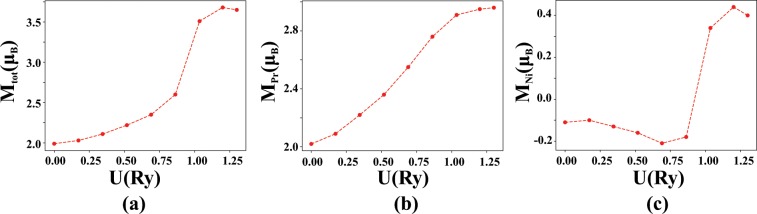
Total (**a**), Pr (**b**) and Ni (**c**) magnetic moments as a function of Hubbard U value.

In order to study the nature of magnetism in this compound, we investigate the effect of applying the Hubbard U value on the total magnetic moment and on the magnetic moments of Pr and Ni atoms. Results are shown in Fig. 7. According to this figure, variation of the total magnetic moment with U is similar to the behavior of the equilibrium lattice parameter. Total magnetic moment increases from 2 *μ*_B_ for U = 0 to the saturated value of 3.6 *μ*_B_ for U = 1 Ry. The magnetic moment of the Pr atom, also, increases from 2.02 *μ*_B_ to 2.92 *μ*_B_, which indicates that the Pr atom has the dominant contribution to the total magnetic moment. As it can be clearly seen from Fig. 5, the Hubbard potential pushes the Pr-f states below the Fermi level and make them more localized, which results in larger Pr magnetic moments.

From Fig. 7 it is clear that the magnetic moment of the Ni atom behaves differently, as the Hubbard U potential increases. At First, the Ni magnetic moments are oriented opposite to the Pr magnetic moments with the value of −0.11 *μ*_B_. Afterwards, by increasing U, they reorient parallel to the Pr magnetic moments and finally, reach to the value of 0.4 *μ*_B_. The magnetic moment of Ni for U = 0.95 Ry is equal to zero. Based on the band structure diagrams, the reason is that by applying U, the bondings between Pr-f states and other orbitals, specially, with the Bi-p states are broken and the position of the Ni-d states change.

### AFM calculations

**Figure 8:**
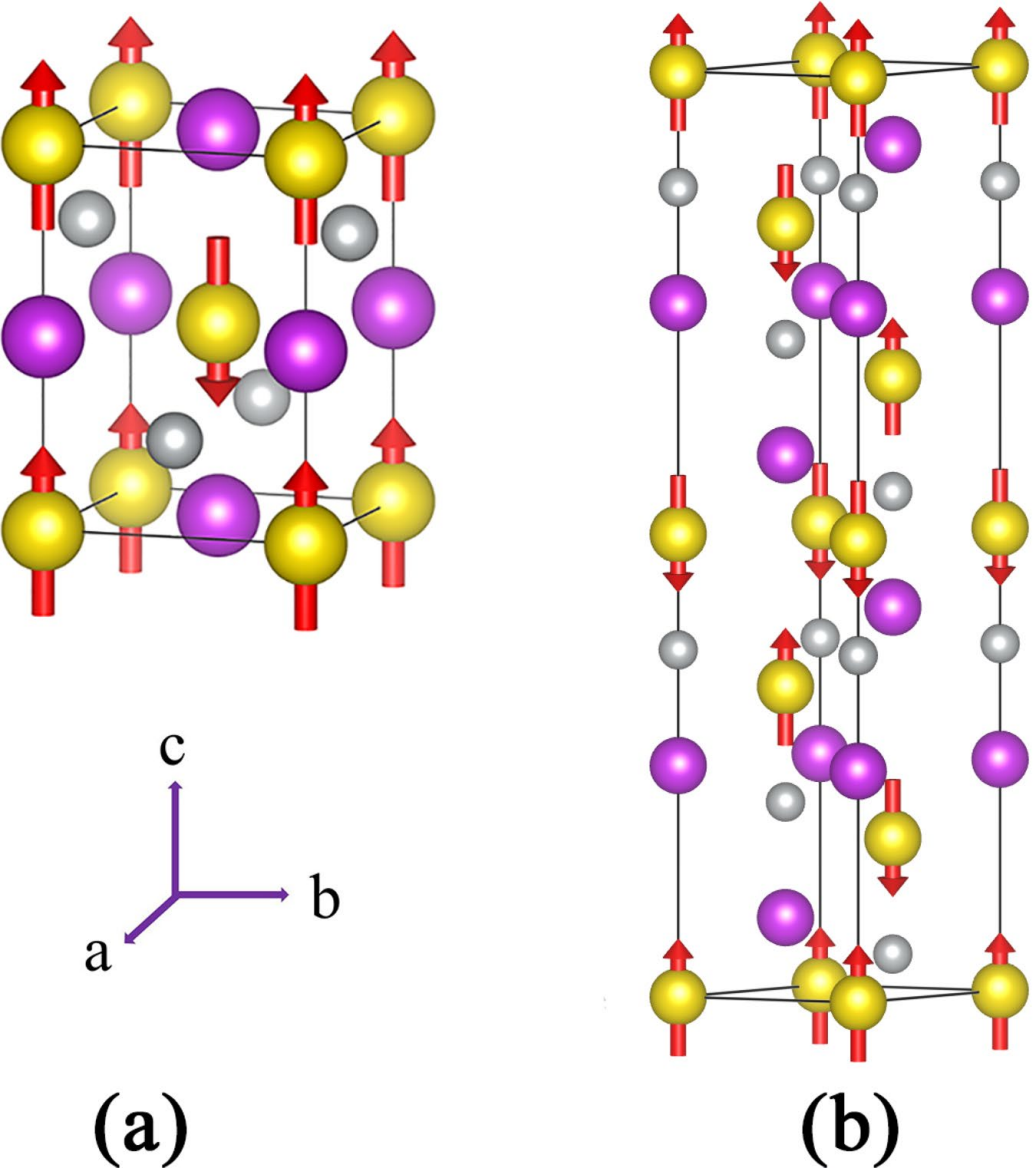
Schematic demonstration of two AFM magnetic orderings, tetragonal (**a**) and orthorhombic (**b**) in PrNiBi. Yellow, purple and gray balls represent Pr, Bi and Ni atoms, respectively.

Given that the low temperature state of PrNiBi is AFM with *T*_*N*_ = 10*K*, we continue the calculations by considering AFM ordering for the magnetic moments of the Pr atoms. Because there is no detailed experimental data on the magnetic ordering of the Pr atoms in PrNiBi, we consider two most probable magnetic configurations that were also suggested for other similar half-Heusler compounds. In the first AFM ordering (AFM-I), which is observed in the GdPtBi system^51^, the Pr magnetic moments are aligned ferromagnetically in the (111) plane and are stacked antiferromagnetically perpendicular to these planes. Considering this magnetic ordering, the crystal symmetry is decreases and one finds an orthorhombic magnetic unit cell with *R*3*m* space group as depicted in Fig. 8(b). The second AFM ordering (AFM-II) has been reported in the NdPtBi and CePtBi compounds^52^, where the ferromagnetic planes are stacked antiferromagnetically along the propagation vector [100]. The smallest unit cell of this magnetic ordering is tetragonal as depicted in Fig. 8(a), with *Pmm*2 space group. Both AFM structures undergo a spin flip when they are displaced by the translation vector *D* = (1/2,1/2,1/2), which can preserve the Kramer’s degeneracy^49^, which is necessary for a spin quantum Hall system.

For all AFM calculations, equilibrium lattice parameters are obtained which are almost equal to the FM values. In AFM-I structure without applying the Hubbard U, the magnetic moment of Pr atom is obtained equal to 2.03 *μ*_B_ and the Ni magnetic moment is negligibly small. Similar to the FM calculations, Bi-p bands are formed 0.5 eV below and Ni-s states are formed 0.8 eV above the Fermi level, at the Γ point.

**Figure 9:**
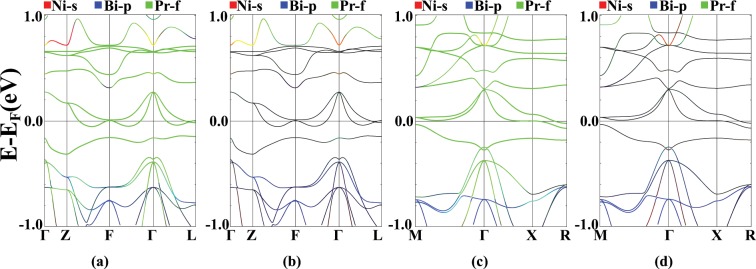
Band structure plots for AFM-I (**a**) majority and (**b**) minority spin channel, and AFM-II (**c**) majority and (**d**) minority spin channel of PrNiBi, with the characterization of the Ni-s (red), the Bi-p (blue) and the Pr-f (green) states. Calculation are performed for relaxed lattice parameters.

The band structures of AFM-I and AFM-II are shown in Fig. 9. In the spin majority channel, Pr-f bands form in the range of −0.5 to 0.7 eV respect to the Fermi level, and in the spin minority channel they are located at higher energies, about 1 eV above the Fermi energy. Also, The Ni-d states have a strong hybridization with Bi-p states in the range of −0.5 to −3 eV in both spin channels. At energy levels about 0.8 eV above the Fermi level, the Ni-s and the Bi-s orbitals are mixed. A rather weak hybridization can be seen at −0.5 eV, between the Pr-f bands and the Bi-p states. The Ni-d states have a large hybridization with the Pr-f states near the Fermi level. Due to the localized nature of the magnetization in lanthanide elements and the low tendency of f states to hybridize with other atomic orbital, it is necessary to apply the Hubbard on-site potential to correct the position of f bands, in this system. Applying U, up to the values of 0.7 Ry causes the localization of the Pr-f orbitals, and pushes the Bi-p states to higher energy values which results in a s-p band inversion. The hybridization of the Ni-d and the Bi-p states around the Fermi level, is also evident.

**Figure 10:**
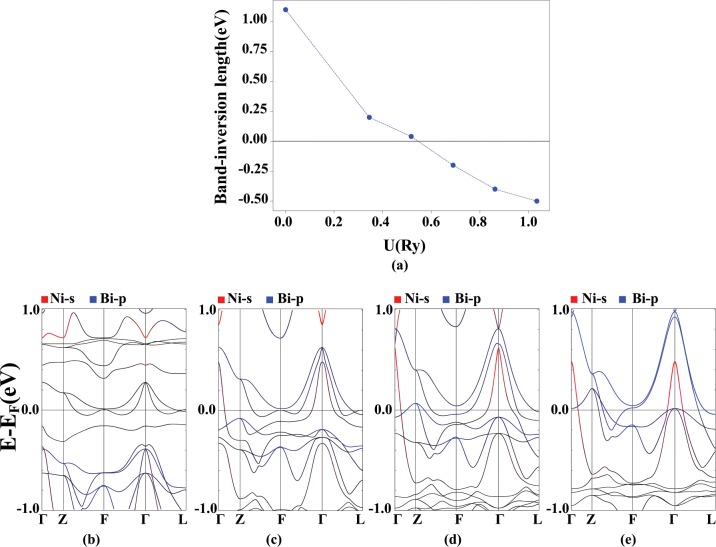
Band inversion strength as a function of U (**a**) and band structure diagrams ((**b**–**e**)) for the AFM-I ordering in PrNiBi with the characterization of the Ni-s (red) and the Bi-p (blue) states, for different values of U = 0, 0.345, 0.69 and 1.035 Ry from left to right. Calculation are performed for relaxed lattice parameters obtained for each U value.

The energy difference between the s and p states at the Γ point is called the band-inversion strength Δ*E* = *E*_*s*_ − *E*_*p*_. Negative values of Δ*E* mean a band inversion that can be a signature of non-trivial topological phase. For the AFM-I configuration, the graph of the band inversion strength in terms of the applied U is plotted in the Fig. 10(a). Also, band structure diagrams for different values of U are plotted in Fig. 10(b–e). By applying U, f orbitals are pushed below from the Fermi level. Bi and Ni states approach to each other for U = 0.5 Ry. For higher values of U, Ni-s states move to lower energies respect to the Bi-p states, resulting in a band inversion. In the band structure, the Pr-d states seem to contribute in the magnetic exchange between the Pr-f electrons, which are strongly mixed with Bi-p and Ni-d states.

**Figure 11:**
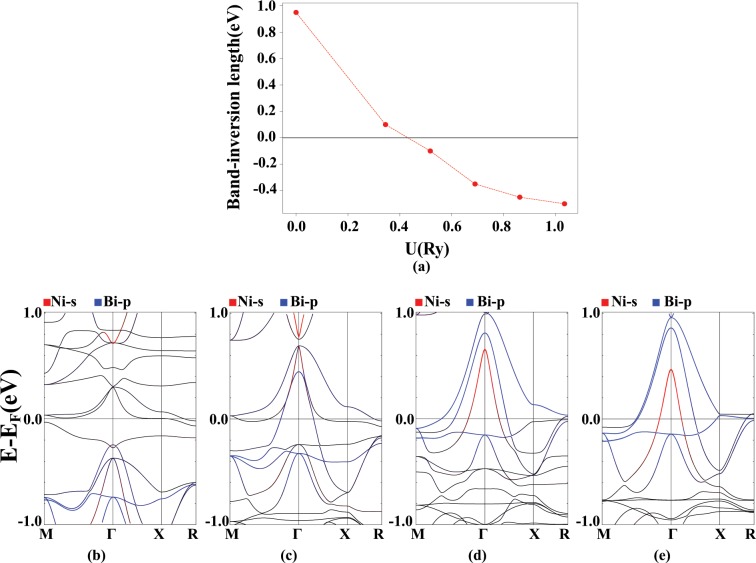
Band inversion strength as a function of U (**a**) and band structure diagrams (**b**–**e**) for the AFM-II ordering in PrNiBi with the characterization of the Ni-s (red) and the Bi-p (blue) states, for different values of U = 0, 0.345, 0.69 and 1.035 Ry from left to right. Calculation are performed for relaxed lattice parameters obtained for each U value.

For the AFM-II phase, the diagram of band inversion strength versus U is plotted in the Fig. 11(a). In Fig. 11(b–e), the band structure diagrams for different values of U are plotted which are almost identical with the AFM-I ordering.

**Figure 12:**
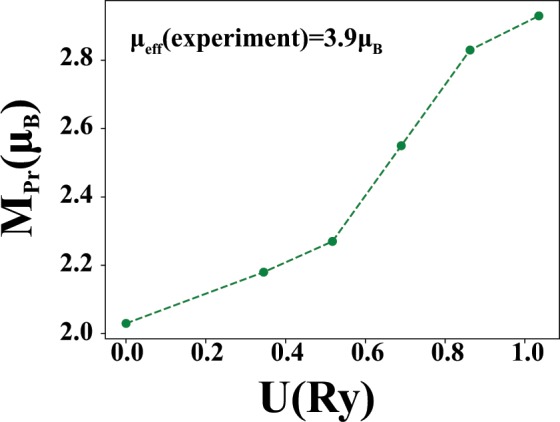
The magnetic moment of the Pr atom in terms of the U value in both AFM configuration.

Changes in the magnetic moment of the Pr atom respect to the value of U for both AFM phases are shown in the Fig. 12. The results are similar that of the FM calculation, i.e. Figure 7. The saturation moment is equal to 2.9 *μ*_B_. Experimental measurements have reported an effective Pr magnetic moment equals to 3.9 *μ*_B_^55^, in this compound. This shows that a reasonable calculation cannot be done while ignoring the effects of on-site electron interactions. The only notable difference between the AFM-I and the AFM-II phases is in the magnetic moments of the Ni atoms, which in both of the phases is smaller than the FM case. In the AFM-I state, for large values of U, the magnetic moment of Ni reaches to 0.16 *μ*_B_. In the AFM-II configuration, the obtained value for the Pr magnetic moment is similar to the AFM-I case but the Ni is non-magnetic.

According to the band structure diagrams and the calculated magnetic moments, it is possible to explain the magnetic interaction in this compound. Unlike 3d-transition metals, in rare earth elements, f states remain highly localized leading to localized magnetic moments. In this compound, a direct exchange interaction exists between the Ni atoms due to the strong overlap of the 3d-electron wave-functions. Investigating the spin polarized charge densities reveals that the electron spins in the vicinity of Pr atoms are polarized and aligned parallel to the spins of the Pr-4f electrons, due to the local magnetic field. The magnetic exchange between different Pr sites is mediated by those electrons. Most noticeably, a slightly filled 5d band plays the important role in the exchange mechanism between the Ni-3d and Pr-f magnetic moments.

**Figure 13:**
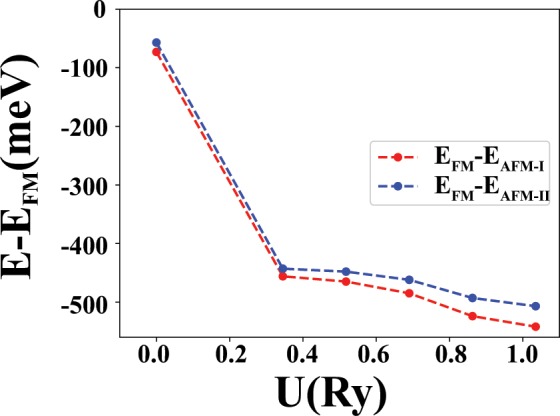
Energy difference between the FM and the two AFM configurations.

Finally, in order to study the phase stability among different magnetic configurations, the energy difference between the FM state with two AFM states is plotted in Fig. 13, in terms of the Hubbard U. For all U values, the AFM configurations have lower energies respect to the FM state and the AFM-I phase is more stable than the AFM-II phase. As a result, the AFM-I phase is the native phase of this compound, ignoring the possibility of more complex AFM orderings.

### Effect of SOC

**Figure 14:**
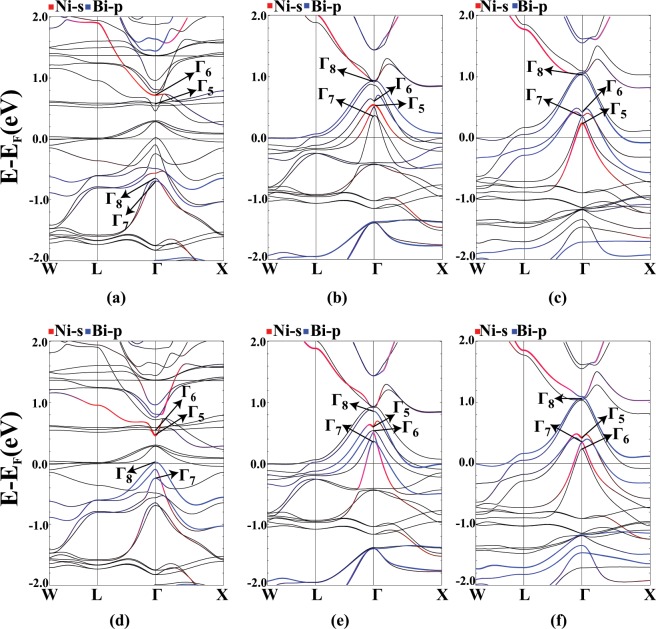
FM, *σ*GGA + U band structures for different values of U = 0, 0.69 and 1.035 Ry from left to right, in majority (top row) and minority (bottom row) spin channels, with Ni-s (red), Bi-p (blue) orbitals characterization with soc. Calculation are performed for relaxed lattice parameters obtained for each U value.

**Figure 15:**
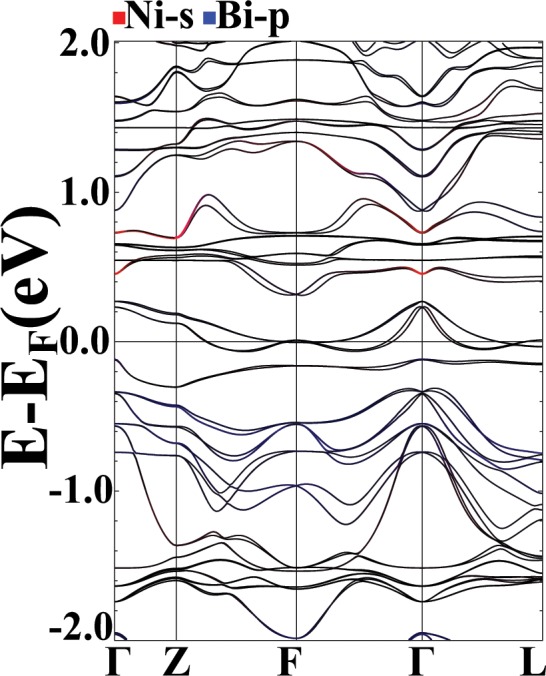
AFM band structures with Ni-s (red), Bi-p (blue) orbitals characterization with soc. Calculation are performed for relaxed lattice parameters.

In order to justify the discussed band inversions in the *PrNiBi* alloy, here, we turn on the SOC and investigate the bands at the Γ-point. Calculated band structure for the FM system, including SOC and Hubbard-U interaction with U = 0,0.69, 1.035 Ry is depicted in Fig. 14. The triply-degenerated Γ_5_ p-states of Fig. 5, with *Bi* − *p* character, splits into Γ_8_ and Γ_7_ states and the s-like Γ_1_ state of that figure, splits into Γ_5_ and Γ_6_ states. By comparing Figs. 5 with 14, it is clear that including SOC, does not significantly change the position of the Γ_8_ states and one can study the Γ_6_–Γ_8_ band inversion qualitatively by inspecting the band structures without SOC. In Fig. 15 the calculated band structure for the AFM system, including SOC is depicted. Again, a comparison with Fig. 9(a,b) indicates that despite the basic necessity of SOC for a topologically non-trivial state, it usually does not alter the order of bands^61^ and the role of other electronic interactions are dominant.

## Summary and Conclusion

In summary, we have performed *ab initio* DFT calculations using the *σ*GGA + U method for the PrNiBi half-Heusler alloy. An AFM ground state is obtained in agreement with the experiment in which the estimated magnetic moments of the Pr atoms is equal to 2.9 *μ*_B_. The saturated magnetic moment of the Pr atom is close to the experimental values only when the on-site electron interaction U is applied. Also, a s-p band inversion is found in the band structure diagrams by applying the U interaction, which can be a signature of a topologically non-trivial state.
